# The impact of physiological loading on immune cell infiltration and myocardial function evaluated by cardiac MRI: a comparison between non-working heart and working heart transplant models

**DOI:** 10.1186/1532-429X-14-S1-P220

**Published:** 2012-02-01

**Authors:** Qing Ye, Yi-jen L Wu, Yeh Fang-Cheng, Li Liu, Brent Barbe, Lesley M Foley, T Kevin Hitchens, Chien Ho

**Affiliations:** 1 Pittsburgh NMR Center for Biomedical Research, Carnegie Mellon University, Pittsburgh, PA, USA; 2 Department of Biomedical Engineering, Carnegie Mellon University, Pittsburgh, PA, USA; 3 Department of Biological Sciences, Carnegie Mellon University, Pittsburgh, PA, USA

## Summary

Rodent models of heterotopic cardiac transplantation are important for gaining a better understanding of heart allograft rejection. This study compared a working heart (WkHt) transplant model, which have developed by our laboratory, with the traditional non-working heart (Non-WkHt) model using cardiac MRI (CMRI) and pathological evaluation. Our results indicate that the physiological hemodynamic loading significantly impacts graft immune cell infiltration and myocardial function. Our WkHt transplant model with intact pulmonary circulation, retains hemodynamic loading close to that of native heart, and is thus more appropriate for both functional and immunological studies, especially for developing the clinically relevant CMRI techniques for comprehensive evaluation of the heart graft.

## Background

The traditional heterotopic heart transplantation rodent model [1,2] lacks blood flow toward left ventricle (LV) and the graft does not pump blood, and therefore is a non-working heart (Non-WkHt). We have developed a new heterotopic working heart (WkHt) transplant model with intact pulmonary circulation to study cardiac allograft rejection.[3] In this study, we used combinations of pathology and multi-parameter CMRI, such as cine, Tagging, and T1/T2, to comprehensively evaluate the impact of physiological loading on the myocardial function, immune cell infiltration and graft pathophysiological condition of these 2 models.

## Methods

WkHt model: En bloc of graft heart and lung was transplanted into the recipient abdomen. Graft aorta and superior vena cava (SVC) were anastomosed with recipient abdominal aorta and inferior vena cava (IVC), respectively.[3,4] Non-WkHt model: The graft aorta and pulmonary artery were anastomosed with the recipient abdominal aorta and IVC, as previously described.[1,2] CMRI: Cine and tagging MRI were used to assess cardiac function, and cellular MRI was performed using T2*-weighted scans to detect in situ micro-meter sized iron (MPIO) labeled macrophage infiltration at 7 Tesla. At the end point hearts were perfused and fixed for MR microscopy (MRM) at 11Tesla and pathological examination.

## Results

**Figure 1:**
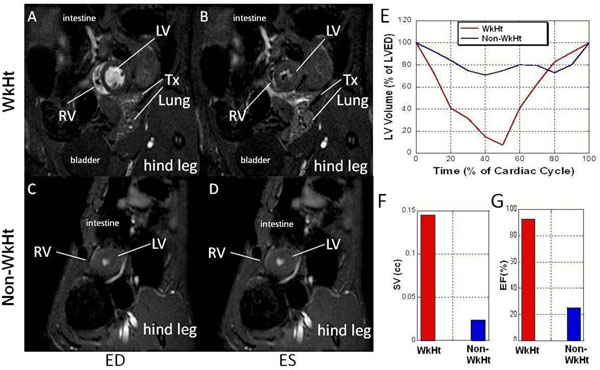
Cardiac functions of isograft hearts from 2 HTx models evaluated by cine MRI. (A, B) cine imaging of an isograft from the WkHt model; (C, D) cine imaging of an isograft from the Non-WkHt model; (A, C) cine images acquired at end-diastole (ED); (B, D) cine images acquired at end-systole (ES); (E) temporal dynamic of normalized left ventricular volume over a cardiac cycle, (F) SV, and (G) EF, of a WkHt (red) and a Non-WkHt (blue).

**Figure 2:**
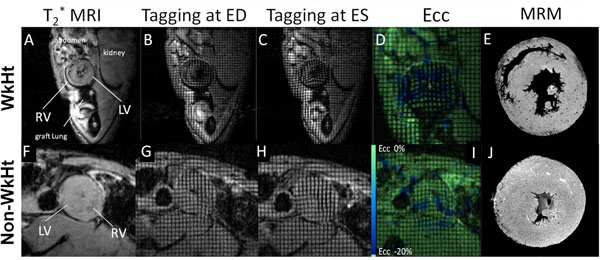
CMRI of allografts hearts from the WkHt model on POD 47 (top panels, A-E) and the Non-WkHt model on POD 41 (lower panels, F-J). (A, F) in vivo T2* MRI; (B, G) tagging MRI at ED; (C, H) tagging MRI at ES; (D, I) circumferential strain (Ecc) maps derived from tagging MRI; and (E, J) ex vivo T2* MR Microscopy (MRM). The hypointensity regions/sports appeared in A, F, E and J result from MPIO-labeled monocytes/macrophages.

Although isograft hearts from both models had strong heart beats and normal ECG, they exhibited very different cardiac functions revealed by CMRI (Fig.1). The WkHt displayed filling and ejecting cardiac phases (Fig.1A, B, E) comparable to native hearts. The graft stroke volume (SV, Fig.1F) is near to normal condition, and the ejection fraction (EF) was greater than 90% (Fig. 1G). On the contrary, the isograft from Non-WkHt model showed very little filling and ejecting (Fig.1C, D, E ), and its SV (Fig. 1F) and EF (Fig. 1G) are significantly lower than the WkHt. A similar functional discrepancy can also be seen in allograft hearts (Fig.2) but the degree is compounded by allograft rejection. Tagging MRI (Fig.2B, C, G, H) and strain analysis (Fig.2D, I ) showed that allograft heart from WkHt model preserved much more wall motion than Non-WkHt. Interestingly, immune cell infiltration was also affected by the model choice. T2*-weighted MRI with MPIO-labeled macrophages showed different infiltration pattern in two models (Fig. 2A, F, E, J).

## Conclusions

Our results indicate that hemodymamic loading significantly impacts the graft status. The WkHt transplant model, with intact pulmonary circulation, retains physiological conditions close to that of the native heart, and therefore is more appropriate for functional and immunological studies, especially for developing the clinically relevant CMRI techniques for comprehensive evaluation of the heart graft.

## Funding

This study is supported by grants from the National Institutes of Health (P41EB001977 and R01HL-081349).
